# Thyroid-stimulating hormone-secreting pituitary adenoma presenting with recurrent hyperthyroidism in post-treated Graves’ disease: a case report

**DOI:** 10.1186/1752-1947-7-27

**Published:** 2013-01-21

**Authors:** Yoshikazu Ogawa, Teiji Tominaga

**Affiliations:** 1 Department of Neurosurgery, Kohnan Hospital, 4-20-1 Nagamachiminami, Taihaku-ku, Sendai, Miyagi, 982-8523, Japan; 2 Department of Neurosurgery, Tohoku University Graduate School of Medicine, Sendai, Japan

**Keywords:** Graves’ disease, Pituitary adenoma, Sequential occurrence, TSH

## Abstract

**Introduction:**

The coexistence of autoimmune hyperthyroid disease and thyroid-stimulating hormone-secreting pituitary adenoma is rare. The simple presumption of coincidence of these two diseases has a calculated incidence of less than one/several hundred million, and only four cases with histological confirmation have been reported. A rapid decrease in thyroid-stimulating hormone level after pituitary tumor removal may induce subsequent activation of autoimmune responses against the thyroid gland. We report the first case of a sequential and paradoxical occurrence of Graves’ disease and a thyroid-stimulating hormone-secreting pituitary adenoma.

**Case presentation:**

A 32-year-old Japanese woman had recurrent hyperthyroidism. She had a history of Graves’ hyperthyroidism, which had been successfully treated with propylthiouracil. A head magnetic resonance imaging showed a less enhanced area in the left lateral wing of her sella turcica. Transsphenoidal surgery was performed, and the diagnosis was established as thyroid-stimulating hormone-secreting plurihormonal adenoma. A rapid reduction in thyroid hormone levels was achieved, and her blood pressure was normalized after the operation.

**Conclusion:**

Although incidental occurrence is the most probable etiology, long and repeated followup examinations of both thyroid and pituitary gland should be performed in patients with an atypical clinical course.

## Introduction

Autoimmune thyroid disease coexisting with thyroid-stimulating hormone (TSH)-secreting pituitary adenoma is rarely reported [1]. Postmortem study of 64 patients with presumed primary hypothyroidism demonstrated the presence of 12 adenomas in 10 patients, five of which were positive for TSH staining [2]. Induced hypothyroidism progressed to focal adenoma within about six months in a mouse experimental model [3,4]. However, most cases describe the coexistence of hypothyroidism and a TSH-secreting pituitary adenoma. Only four cases of coexistence with hyperthyroidism have been reported [5-8], and three of the four patients developed Graves’ disease after removal of the TSH-secreting pituitary adenomas [5-7]. Graves’ disease preceding the occurrence of a TSH-secreting pituitary adenoma has never been described. We report a patient with Graves’ disease causing recurrent hyperthyroidism who became euthyroid, but subsequently developed a TSH-secreting pituitary adenoma.

## Case presentation

**Figure 1:**
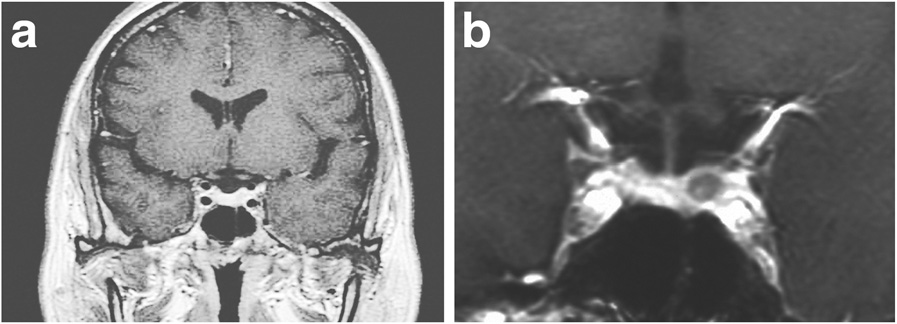
Initial coronal (a) magnetic resonance imaging showing normal appearance of the sella turcica and pituitary gland, and follow-up coronal (b) magnetic resonance imaging showing a less enhanced area in the left lateral wing of the sella turcica.

**Figure 2:**
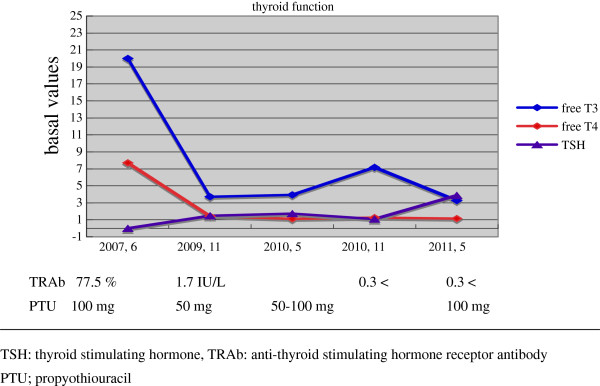
**Serial evaluation of pituitary thyroid hormones.** PTU: propylthiouracil, TRAb: anti-thyroid stimulating hormone receptor antibody,TSH: thyroid-stimulating hormone.

A 32-year-old Japanese woman with recurrent hyperthyroidism was introduced to the out-patient department of Kohnan Hospital. She had a family history of Graves’ disease. She began to feel thirst, frequent palpitations, and body weight loss from around June 2006, and hyperthyroidism was detected. Her serum free triiodothyronine (T3) was more than 20pg/mL, free thyroxine (T4) was 7.7ng/dL, TSH was less than the detectable level and anti-TSH receptor antibody (TRAb) was 77.5% (normal range, less than 10%). The 24-hour uptake of iodine-123 to the thyroid gland was 38.47% in the right lobe and 44.16% in the left lobe (total 82.63%). A head magnetic resonance imaging (MRI) with contrast medium revealed thickening of the bilateral ocular muscles, but no evident tumor in the sella turcica (Figure 1a). The diagnosis was established as Graves’ disease, and propylthiouracil (PTU) was administered. TRAb had fallen to the normal range, and a euthyroid state was achieved in June 2008. However, occasional elevation of free T3 was detected, so PTU administration was continued. Re-elevation of TSH was seen in December 2010 (Figure 2). A head MRI revealed abnormal findings in the sella turcica, and she was introduced to the neurosurgical department of Kohnan Hospital in May 2011.

On admission her blood pressure was 152/112mmHg. Electrocardiography recorded her heart rate as 70 beats/minute but she complained of frequent palpitations. Her consciousness was clear, and no abnormal neurological signs were detected. Endocrinological examinations in the morning with oral intake of 100mg of PTU revealed free T3 of 2.77pg/mL, free T4 of 1.09ng/dL and TSH of 2.433μIU/mL. Because of her frequent palpitations the thyroid-releasing hormone (TRH) loading test without discontinuing PTU was performed, which revealed no abnormal response. All other pituitary hormones were within the normal ranges, and head MR imaging with contrast medium showed an area of less enhancement in the left lateral wing of the sella turcica with a diameter of 5mm (Figure 1b), and transsphenoidal surgery was planned under a diagnosis of TSH-secreting pituitary adenoma.

**Figure 3:**
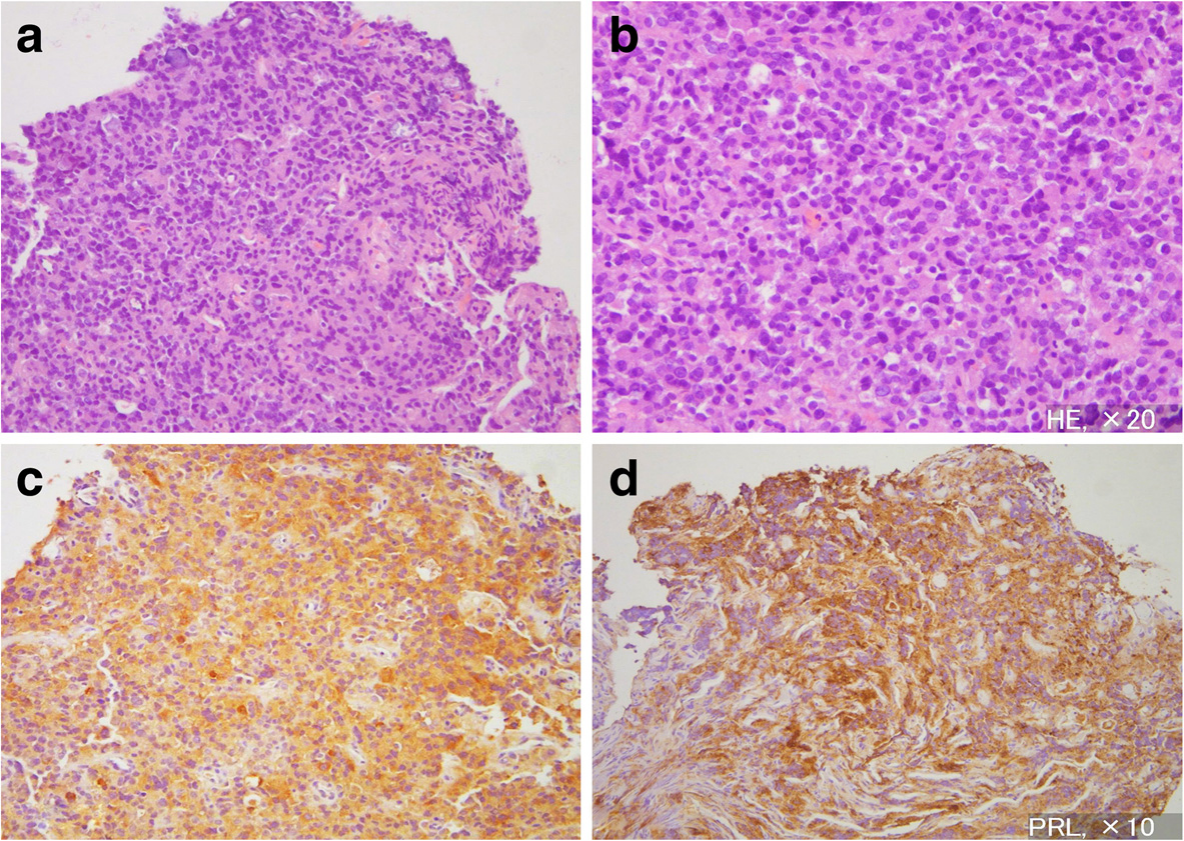
**Photomicrographs of the surgical specimen of the sellar tumor showing diffuse cell arrangement with mild variation in size but without atypism or mitosis of the nucleus.** Hematoxylin and eosin staining, original magnification × 40 (**a**), × 80 (**b**), and immunohistochemical staining for anti-thyroid-stimulating hormone-β (**c**), PRL indicates anti-prolactin (**d**) antibody. Original magnification × 40. Number indicates magnification of the objective lens.

The milky-white soft tumor was enclosed within a thin cellulose-like membrane. Total removal was achieved in addition to medical fixation of the cleavage with pure ethanol. Postoperative histological examination showed diffuse cell arrangement with mild variation in size but without atypism or mitosis of the nucleus (Figure 3a,b). Immunohistochemical examination disclosed plurihormonal expression of pituitary hormones including TSH-β, and the diagnosis was established as TSH-secreting plurihormonal adenoma (Figure 3c,d).

Administration of PTU was discontinued from the day of surgery. Diastolic blood pressure did not exceed 100mmHg for six days after the operation. Pituitary and thyroid hormones were re-evaluated at 11 days after the operation, showing free T3 was 3.31pg/mL, free T4 was 1.00ng/dL and TSH was 1.567μIU/mL. Additional TRH loading test found no abnormal responses, and she was discharged at 12 days after the operation. Her blood pressure was 133/86mmHg, and palpitations had disappeared without medication.

## Discussion

Graves’ disease induces hyperthyroidism, principally in women aged over 20 years with an annual incidence of 0.5/1000 populations [1]. TSH-secreting pituitary adenoma accounts for around 1% of all pituitary adenomas and has an annual incidence of 1–8/10,000,000 [9]. Therefore, the simple presumption of coincidence of these two diseases has a calculated incidence of less than one/several hundred million, and only four cases with histological confirmation have been reported [5-8]. Three of these four cases developed after removal of the TSH-secreting pituitary adenomas [5-7]. Our patient presented with progressive Graves’ disease, but TSH-secreting adenoma only developed after a euthyroid state was achieved. There is no theoretical explanation for these two pathogeneses. Anti-thyroid medication administered under a misdiagnosis of Graves’ disease may carry the risk of promotion of TSH-secreting pituitary adenoma due to the positive feedback system [10]. Our patient had elevation of TRAb, excessive relative uptake of radioactive iodine to the thyroid gland and absence of pituitary tumor on head MRI, which satisfy the diagnostic criteria of Graves’ disease. We could not exclude the possibility of extremely minute pituitary adenoma, undetectable by MRI, which might be stimulated by PTU administration resulting in the sequential occurrence of TSH-secreting adenoma. However, incidental occurrence was considered to be the most probable explanation.

Diagnosis of TSH-secreting pituitary adenoma is sometimes very complicated. Recent advances in a highly sensitive immunoassay for TSH are expected to improve the accuracy of diagnosis. However, if inappropriate TSH secretion is very mild, the TSH level remains within the normal range because of the negative feedback system to prevent excessive secretion of thyroid hormones. Measurement of the molar ratio of serum α-subunit to TSH and assessment of response to the TRH loading test might be useful, but commercial kits for an assay of the former have been discontinued in Japan since the beginning of 2011. These factors should be systemically evaluated.

## Conclusion

We report a unique case of recurrent hyperthyroidism preceding the occurrence of TSH-secreting pituitary adenoma in a patient who became euthyroid after treatment for Graves’ disease. Although incidental occurrence is the most probable etiology, we emphasize the importance of repeated followup examinations of both thyroid and pituitary gland in patients with an atypical clinical course.

## Consent

Written informed consent was obtained from the patient for publication of this manuscript and accompanying images. A copy of the written consent is available for review by the Editor-in-Chief of this journal.

## Competing interests

The authors declare that they have no competing interests.

## Authors’ contribution

YO performed tumor removal and analyzed the patient data regarding the endocrinological outcome, and was a major contributor in writing the manuscript. TT gave an essential suggestion and supervised this manuscript. Both authors read and approved the final manuscript.
